# Redefining Carbon Electrodes as Hierarchically Designable Reaction Fields for Electrochemical Biosensing

**DOI:** 10.1002/smll.75692

**Published:** 2026-09-08

**Authors:** Hiroshi Yuzawa, Tetsuji Itoh, Thidarut Laochai, Nadnudda Rodthongkum, Hirotomo Nishihara

**Affiliations:** ^1^ Institute of Multidisciplinary Research for Advanced Materials Tohoku University Sendai Miyagi Japan; ^2^ Department of Chemical Engineering Graduate School of Engineering Tohoku University Sendai Miyagi Japan; ^3^ Research Institute for Chemical Process Technology National Institute of Advanced Industrial Science and Technology (AIST) Sendai Miyagi Japan; ^4^ Department of Materials and Textile Technology Faculty of Science and Technology Thammasat University Pathum Thani Thailand; ^5^ Center of Excellence in Responsive Wearable Materials Faculty of Science Chulalongkorn University Bangkok Thailand; ^6^ Department of Chemistry Faculty of Science Chulalongkorn University Bangkok Thailand; ^7^ Advanced Institute for Materials Research (WPI‐AIMR) Tohoku University Sendai Japan

**Keywords:** carbon‐based biosensors, hierarchically designable reaction fields, interfacial states, structure–function relationships and multimodal sensing

## Abstract

Electrochemical biosensors based on carbon materials have achieved remarkable sensitivity, yet carbon electrodes are still often treated as black‐box conductive supports. In this perspective, we redefine carbon materials as hierarchically designable reaction fields in which defect chemistry, edge‐site termination, pore architecture, and electron‐transport pathways are integrated as explicit design variables, thereby providing a conceptual framework to guide the rational design of biosensor performance. We highlight how emerging analytical tools, including high‐temperature‐range temperature‐programmed desorption and solid‐state NMR, enable direct correlation of carbon structure with interfacial functions relevant to molecular recognition, charge storage, and signal transduction. We further discuss recent studies showing that rational control of particle morphology, active‐site arrangement, and transparent electrode architectures can improve not only sensitivity but also response stability, signal discrimination, and multimodal readout, highlighting a design direction in which biosensor performance emerges from the integrated engineering of reaction fields rather than isolated material properties. This framework shifts carbon‐biosensor development from empirical optimization toward structure‐guided design for reproducible and practical sensing in complex samples.

## Graphical abstract

This Perspective proposes viewing carbon electrodes as hierarchically designable reaction fields for electrochemical biosensing. Crystallinity, heteroatom dopants, edge sites, and pore size are highlighted as key structural variables governing electron transfer, mass transport, biomolecule immobilization, and local concentration. This framework connects carbon structure with biosensor performance, providing a basis for shifting carbon‐based sensing interfaces from empirical optimization toward rational design.

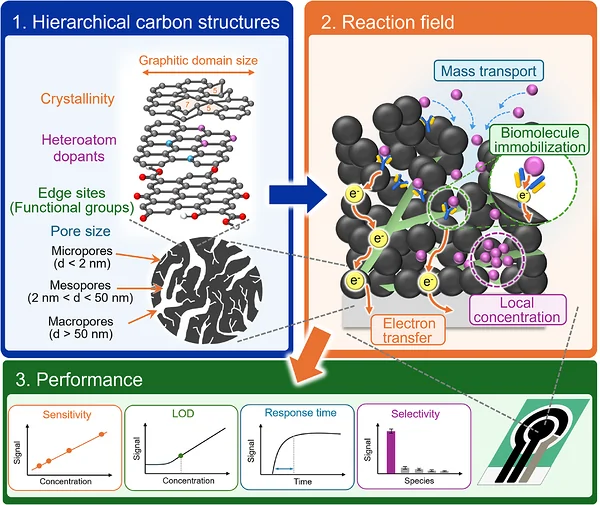

## 1 Introduction

### 1.1 Carbon Electrodes: Widely Used Yet Poorly Defined Design Targets

**FIGURE 1:**
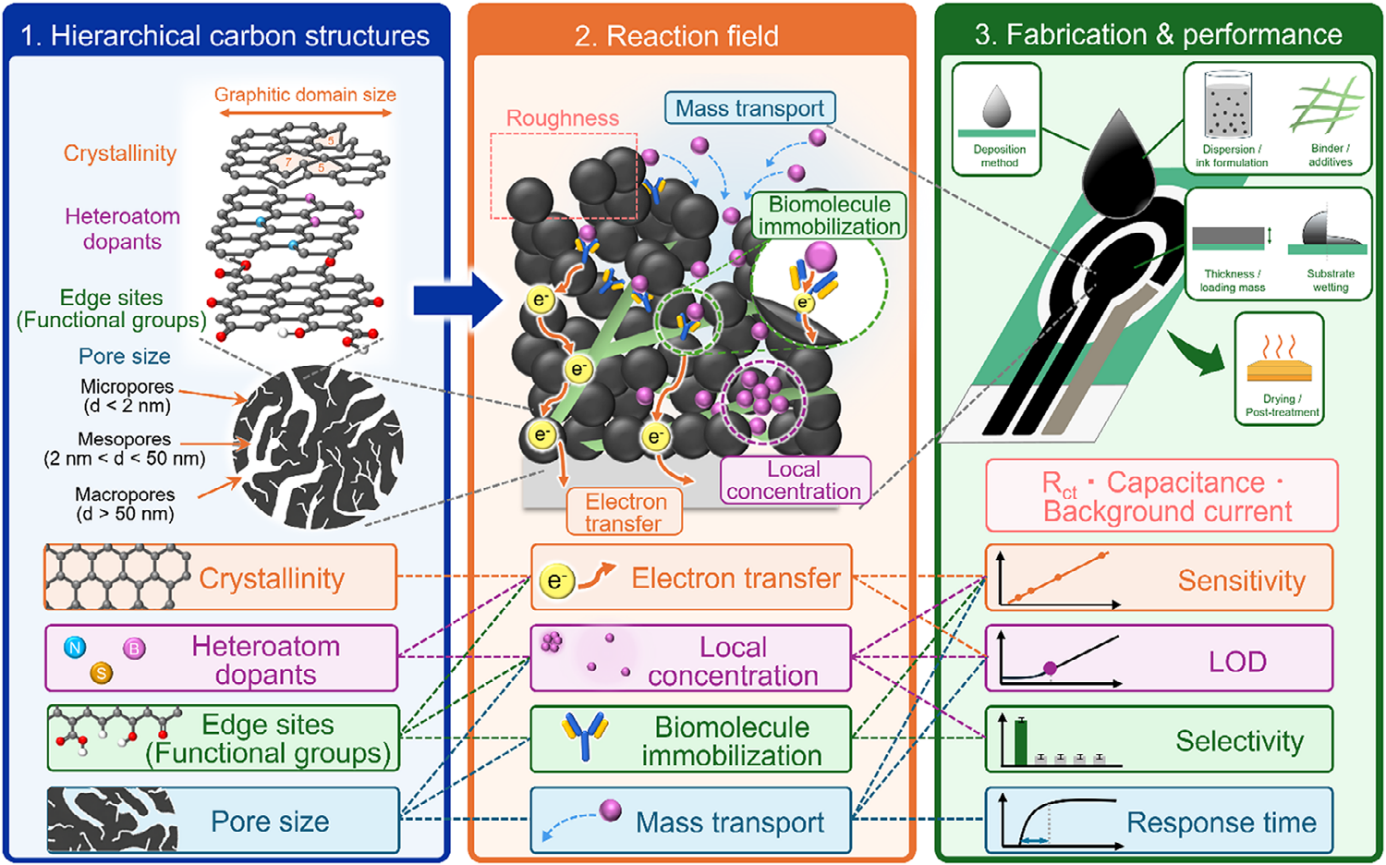
Carbon electrodes as hierarchically designable reaction fields for electrochemical biosensors. Carbon structure and fabrication govern reaction field properties and ultimately determine sensing performance.

Electrochemical biosensors have supported quantitative analysis in medicine and healthcare, food, and environmental applications by converting the molecular recognition of biomolecules into electrical signals [1, 2, 3]. In recent years, advances in materials science, interfacial chemistry, microfabrication, and signal processing have expanded analytes far beyond small molecules to include proteins [4], pathogens [5], and metabolic markers [6], and numerous systems with extremely high sensitivity have been reported. One of the key material classes underpinning this progress is carbon materials. In addition to their high electrical conductivity and chemical stability, carbon materials are lightweight and mechanically flexible, making them well suited for flexible substrates and wearable devices that are difficult to realize with metal electrodes [7, 8, 9]. They can also be readily processed into thin films [10] and inks [11], enabling straightforward electrode fabrication on polymer substrates [12], paper [13], and fibers [11], and making them highly compatible with miniaturized, lightweight, and distributed sensing platforms. Representative carbon materials include graphene, carbon nanotubes, carbon black, and porous carbon, each of which has played a distinct role according to its structural characteristics. For example, graphene has been used as a two‐dimensional carbon material that provides continuous conductive pathways for thin‐film electrodes and composite interfaces [10, 14]. Carbon nanotubes readily form high‐aspect‐ratio networks and have therefore been widely explored for flexible electrodes and printed sensors [11, 15]. Carbon black has been broadly adopted as a practical electrode material because of its low cost, ready availability, and scalability [16, 17], whereas porous carbon [9, 18], including activated carbon [17], has contributed to expanding reaction interfaces through their high surface area. However, most previous uses of carbon materials have focused primarily on improving sensitivity, and the perspective of treating the carbon electrode itself as an explicit design target, including its interfacial state, has not yet been fully established. As a result, carbon materials have often been treated as black boxes, and improvements in practical figures of merit, including the limit of detection (LOD) and response time, have largely depended on empirical optimization of individual experimental parameters such as pretreatment conditions, dispersion conditions, coating thickness, and drying conditions. In this perspective, we redefine carbon materials not simply as highly sensitive electrode materials but as “hierarchically designable reaction fields” (Figure 1). Individual aspects of carbon‐electrode design have already been extensively explored, including defect engineering, surface functionalization, pore‐structure control, printable electrode fabrication, and antifouling interfaces. However, these approaches have largely been developed as separate strategies targeting specific material properties or sensing functions. A unified framework that connects atomic‐scale carbon structure and molecular‐recognition interfaces with electrode‐film formation, sample transport, and the operating environment remains insufficiently established. Here, we integrate these previously fragmented design variables across multiple hierarchical levels, from carbon structure and molecular‐recognition interfaces to electrode formation, sample transport, and operating conditions, and consider their collective influence on the reaction field and biosensor performance. By treating these factors as interconnected design variables, we recast practical figures of merit, including LOD and response time, as problems of reaction field design rather than outcomes of empirical optimization of individual parameters.

## 2 Design Challenges

### 2.1 Why Carbon Electrodes Remain Black Boxes

**FIGURE 2:**
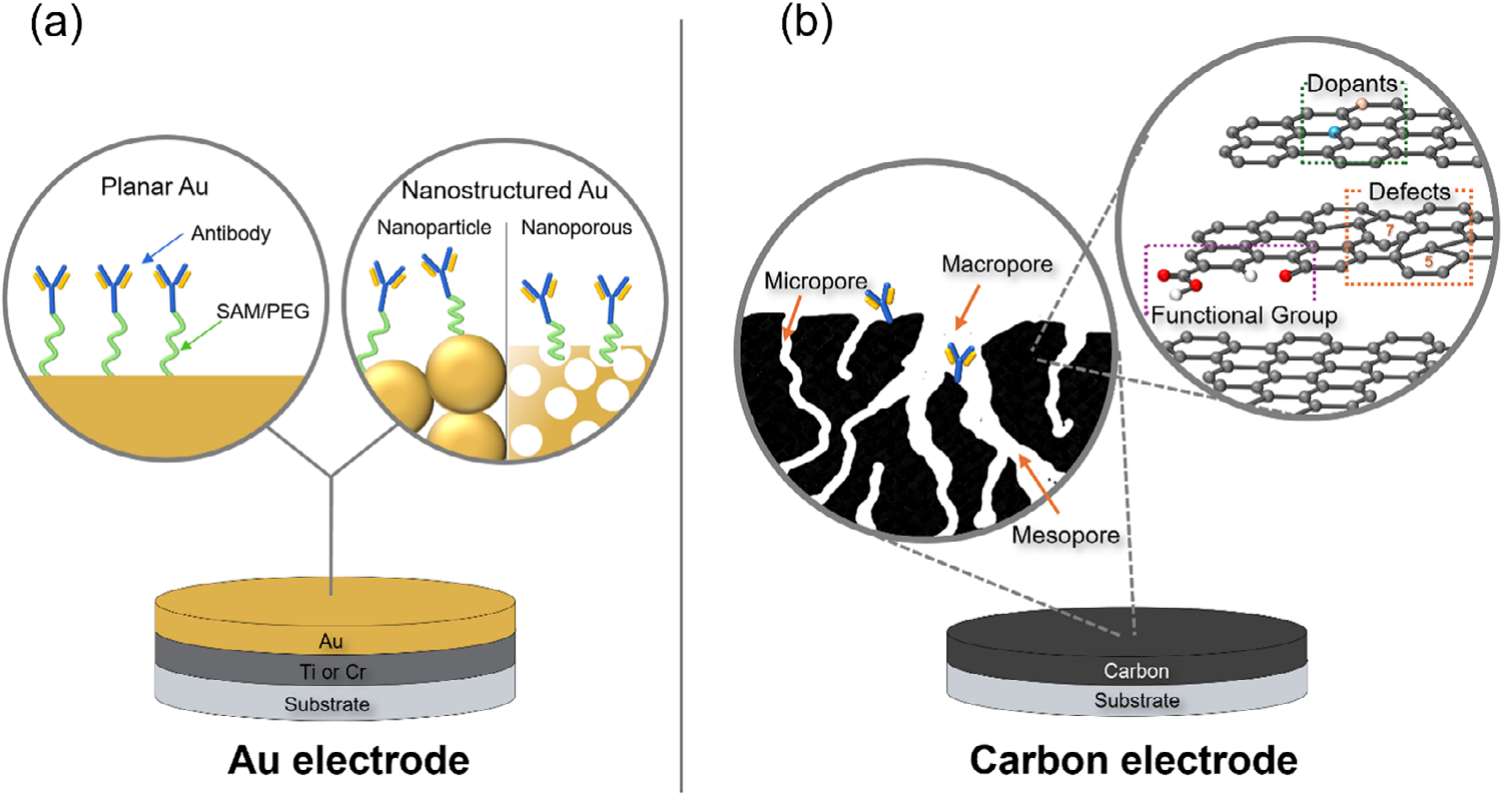
Schematic comparison of Au‐based and carbon electrode design spaces. (a) Planar and nanostructured Au electrodes. (b) Multiscale structural and chemical features of carbon electrodes.

The primary reason carbon electrodes have not been fully treated as explicit design targets in biosensors is that, unlike Au electrodes, carbon materials inherently possess structurally complex and heterogeneous surfaces. Their interfacial states are governed by multiple factors that can simultaneously and intricately influence sensing performance. As a result, even within the same material category, the interfacial state can vary depending on fabrication and pretreatment conditions [19, 20]. The comparison between Au and carbon electrodes is summarized in Table 1 and Figure 2.

**TABLE 1:** Comparison of Au and carbon electrodes in terms of structural and interfacial design opportunities.

Design aspect	Planar Au electrodes	Nanostructured Au electrodes	Carbon electrodes
Interfacial definition	Relatively well‐defined and reproducible surfaces	Dependent on nanostructure morphology and surface state	Often heterogeneous and dependent on structure, fabrication, and pretreatment
Structural design variables	Mainly film thickness and surface roughness	Particle size/shape, porosity, roughness, and facets	Porosity, thickness, graphitic order, defects, edge structures, and dopants
Surface functionalization	Well‐established Au–S chemistry	Au–S chemistry combined with high surface area	Covalent/noncovalent modification through functional groups and π–π interactions
Current practical controllability	Comparatively reproducible using established fabrication and surface‐treatment protocols	Requires reproducible control of both nanostructure and surface chemistry	Strongly dependent on material source, fabrication, pretreatment, and electrode formulation
Potential design space	Mainly surface/interface design	Morphology + surface/interface + composition	Morphology + surface/interface + framework structure + atomic‐scale sites
Major limitations	Contamination, fouling, and stability of surface modification	Structural variability, fouling, and reproducibility of nanostructures	Batch variability, pretreatment and binder effects, surface aging, fouling, capacitive background currents, and film delamination
Key challenge	Maintaining reproducible surface chemistry	Reproducibly coupling nanostructure and surface chemistry	Converting strongly coupled structural and chemical variables into reproducible design rules

Conventionally used planar Au electrodes are typically fabricated by depositing Au, via vacuum evaporation or sputtering, onto glass substrates coated with Ti, Cr, or related adhesion layers. As a result, they present relatively simple and well‐defined surfaces, which can be readily described in terms of crystallographic and chemical states. Such surfaces can be systematically functionalized, for example using thiol‐based self‐assembled monolayers, allowing the incorporation of molecular recognition elements such as antibodies [21], DNA [22] and aptamers [23] (Figure 2a). These characteristics make planar Au electrodes highly suitable for reproducible and predictable interface design. Importantly, however, the design space of Au electrodes is not limited to planar surfaces. Nanostructured Au platforms, including Au nanoparticle‐modified and nanoporous Au electrodes, provide substantial structural and functional tunability through particle size and shape, crystallographic facets, pore structure and surface roughness, accessible surface area, plasmonic properties, and Au–S‐based surface functionalization. Thus, Au electrodes themselves offer considerable flexibility for engineering electrode morphology, interfacial chemistry, and optical properties. Carbon materials provide a complementary but distinct design space. In addition to electrode morphology, pore architecture, and surface functionalization, the carbon framework itself can serve as a design variable through multiple structural and chemical factors, including defect density [24], functional‐group distribution [25], the chemical state of dopants [26, 27], the ratio of basal planes to edge sites [28], and pore architecture [29] (Figure 2b). These features allow carbon electrodes to be engineered not only through electrode morphology and surface chemistry but also through the bonding configuration, electronic structure, and local active‐site environment of carbon framework itself (Table 1). Carbon materials can further incorporate atomically defined active sites, such as metal–N–C motifs, while molecularly derived carbons offer opportunities to embed specific chemical motifs or active‐site precursors within the carbon [30]. Importantly, however, this broad potential design space does not necessarily translate into equally high practical controllability, because these variables are often strongly coupled and sensitive to fabrication and pretreatment. However, this structural diversity also introduces significant challenges. During fabrication and pretreatment, not only the material structure and surface chemistry but also film‐level characteristics, including deposited mass, film thickness and its uniformity, surface coverage, roughness, wettability, adhesion, binder content, and film–substrate interactions, can vary simultaneously. These fabrication‐dependent characteristics determine the actual electrode interface and can thereby alter electron transfer, biomolecule immobilization, mass transport, local concentration, interfacial impedance, and capacitance, ultimately affecting sensor performance (Figure 1). Thus, fabrication and deposition history should be regarded as an integral part of reaction field design rather than merely as experimental conditions. For example, film thickness influences the sensor response through both enzyme loading and mass transport, with excessively thick films potentially limiting analyte transport [31]. Carbon‐ink composition can also alter film formation and electron‐transfer characteristics, thereby affecting sensitivity and LOD [32]. In addition, surface roughness can influence bioreceptor organization and nonspecific adsorption, and consequently the sensitivity and signal‐to‐noise ratio [33]. At the material level, fabrication and pretreatment can simultaneously modify multiple intrinsic carbon characteristics. For example, oxidation simultaneously introduces functional groups and generates defects [34], heat treatment changes crystallinity and surface state [35], and activation modifies pore structure and the local environment at the same time [36]. Consequently, even electrodes prepared from the same nominal carbon material can exhibit different interfacial states and reaction fields, making it difficult to ensure consistent immobilization and reproducible responses even when the same surface functionalization and immobilization conditions are used. In addition, batch‐to‐batch variability, aging of surface functional groups, nonspecific adsorption, fouling, and drift can further alter the interfacial state and electrode response [37]. Capacitive background currents and film delamination can also affect sensor performance, while these dependencies complicate cross‐laboratory standardization. Moreover, even the reference system can affect the performance and lifetime of the working electrode [38]. As a result, sensor performance, including LOD and response time, has depended less on design based on structural factors than on empirical optimization of individual fabrication and pretreatment conditions.

Nevertheless, this structural complexity can be interpreted as a source of design flexibility rather than simply as a source of variability. If these structural and chemical factors together with fabrication‐dependent electrode characteristics can be disentangled and systematically controlled, carbon materials could provide a complementary design framework in which electrode morphology, surface chemistry, electronic structure, and local active‐site environments are jointly engineered to establish the desired reaction field for biosensing.

## 3 Analytical Framework

### 3.1 Defining Carbon by Interfacial State Variables

To exploit the interfacial state of carbon materials in biosensor design, it is first necessary to organize the physical and chemical quantities by which carbon materials have been described. In this section, we focus on analytical methods for describing the atomic and electronic structures and nanoscale structural features of carbon materials. Traditionally, carbon materials have been characterized by average graphene‐sheet size and stacking height estimated by X‐ray diffraction (XRD), defect density and degree of order evaluated by Raman spectroscopy, specific surface area and pore‐size distribution obtained from gas adsorption measurements, and surface functional groups and dopant species analyzed by X‐ray photoelectron spectroscopy and/or FT‐IR. In addition, solid‐state ^13^C NMR has been used to evaluate the sp^2^/sp^3^ ratio, electron spin resonance to assess spin density and unpaired‐electron states, and elemental analysis to determine the overall elemental composition of the material.

**FIGURE 3:**
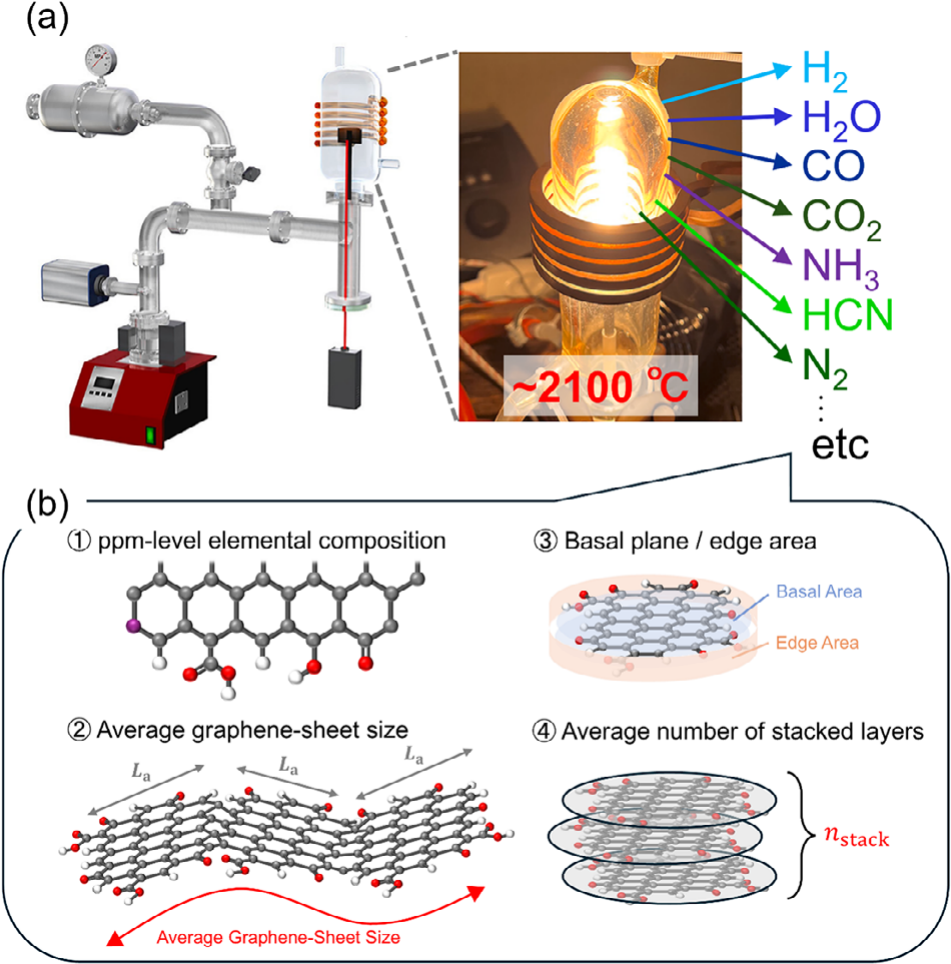
Schematic illustration of (a) the ultra‐high temperature TPD measurement system and (b) the structural descriptors derived from TPD analysis. (a) is reproduced from [27] under CC BY 4.0.

Although these conventional descriptors are indispensable for understanding the structure and composition of carbon materials, they are still insufficient for examining the interfacial functions that support biosensor performance. In particular, the chemical state of edge sites, which act as reaction fields at the electrode interface, provides an important clue that complements conventional descriptors and has attracted attention as a factor strongly influencing the performance and durability of carbon electrodes [39]. In biosensors especially, the adsorption and immobilization states of molecular recognition elements such as enzymes, antibodies, and aptamers govern sensitivity, selectivity, and stability. It is therefore important to understand the density and termination state of edge sites, which serve as interaction points for these species. A technique that can evaluate the chemical state of such edge sites is temperature‐programmed desorption (TPD) [35, 40]. In the TPD analysis, carbon materials are heated in a controlled manner under vacuum or an inert atmosphere with monitoring the desorbed gases. In particular, the desorption amount and temperature distribution of CO and CO_2_ are widely used to analyze corresponding oxygen‐containing functional groups. However, conventional TPD instruments typically operate only up to around 1000°C [41], which limits the quantification of H_2_ species derived from H‐terminated edge sites that desorb at approximately 800–1600°C, making it difficult to obtain a complete picture of edge states. To address this, Kyotani and co‐workers developed high‐temperature range TPD (HT‐TPD), equipped with an induction‐heating system that can operate up to 1800°C under high vacuum (∼10^−5^ Pa) (Figure 3a) [40]. This system enables complete desorption of strongly bound hydrogen species and comprehensive quantification of edge sites.

The HT‐TPD analysis can be extended beyond evaluating the nature of the surface sites where molecular recognition elements adsorb and become immobilized; it can also be used to estimate structural parameters of carbon materials. For example, the average graphene‐sheet size can be estimated even when the sheets are curved, unlike the *L*_a_ value obtained by XRD, which reflects only the size of the planar regions of graphene sheets (Figure 3b) [40]. In addition, HT‐TPD can quantify edge‐site surface area [42], by which basal‐plane surface area can be separated from the total specific surface area obtained from nitrogen adsorption/desorption [43]. Moreover, the precise average number of stacked layers can be estimated by comparing the basal‐plane surface area with the theoretical specific surface area of monolayer graphene [44]. Furthermore, HT‐TPD has been extended to enable qualitative and quantitative analysis of nitrogen species included in carbon materials with high sensitivity at the level of several tens of ppm [27]. HT‐TPD is therefore a highly powerful structural characterization tool that provides access to fundamental structural parameters of carbon materials that are difficult to obtain using conventional analytical methods.

On the other hand, to understand the function of biosensor interfaces, it is important not only to identify local structural and chemical states but also to focus on charge‐storage properties at the interface, namely capacitance. Capacitance influences the ease of charge accumulation at the electrode interface and the magnitude of potential changes, and can therefore affect the sensitivity of signal transduction associated with molecular recognition, background current, and response stability. Alongside the immobilization characteristics of molecular recognition elements, it is thus an important factor in the design of biosensor electrodes. Nanoporous carbons have attracted attention as electrode materials for biosensors because they possess large specific surface areas capable of supporting molecular recognition elements and can also contribute to stabilizing enzymes immobilized inside pores [45]. Conventionally, the capacitance of such nanoporous carbons has been discussed mainly in relation to specific surface area and pore‐size distribution, with the aims of understanding the underlying mechanism and developing guidelines for improvement and control.

In contrast, Alexander C. Forse and co‐workers focused on chemical shifts obtained by solid‐state NMR spectroscopy and introduced “ordered domain size” as a new design factor for carbon frameworks, showing that it correlates with capacitance [46]. In the NMR spectra of carbon materials impregnated with an electrolyte, separate resonances are observed for “in‐pore” ions adsorbed inside carbon nanopores and “ex‐pore” ions located outside the carbon pore network. The in‐pore resonance appears at a lower chemical shift than that of the corresponding free electrolyte because it is influenced by the ring current generated by the circulation of delocalized π electrons in aromatic carbon rings under an applied magnetic field. This shift difference is quantified as a value defined by the chemical shift of the free electrolyte and that of the in‐pore resonance. This value reflects the magnitude of the ring‐current effect and is related to the lateral extent of the aromatic carbon framework forming the carbon pore walls, that is, the average size of graphene‐like fragments. Forse and co‐workers found that the capacitance of carbon materials correlates more strongly with this value than with pore size itself, indicating that the ordered domain size of the carbon framework is a key structural factor governing electrochemical properties. This suggests that in carbons with smaller ordered domains, charge distribution becomes more localized, strengthening interactions between ions and the carbon framework and thereby enabling more efficient ion accumulation.

Thus, although carbon materials have traditionally been treated as black boxes because of their complex internal structures, advances in analytical techniques are beginning to make it possible to capture their structure from both the edge‐site and carbon‐framework perspectives. Both HT‐TPD and solid‐state NMR are emerging analytical techniques that provide descriptors associated with the structural disorder of carbon materials. Recently, efforts have begun to integrate these methods to achieve a more comprehensive understanding of carbon structures and to explore their relationships with electrochemical responses [47, 48].

However, HT‐TPD and solid‐state NMR require specialized instrumentation, which currently limits their routine accessibility, and the relationships between the structural descriptors obtained by these techniques and carbon structure or electrochemical properties have not yet been fully established. As these relationships become better understood, correlations with more widely accessible metrics, such as those obtained from Raman spectroscopy, XPS, XRD, gas adsorption, and electrochemical capacitance measurements, may enable the structural information derived from advanced characterization to be translated into more practical design descriptors.

## 4 Architected Carbon Electrodes for Biosensing Interfaces

**FIGURE 4:**
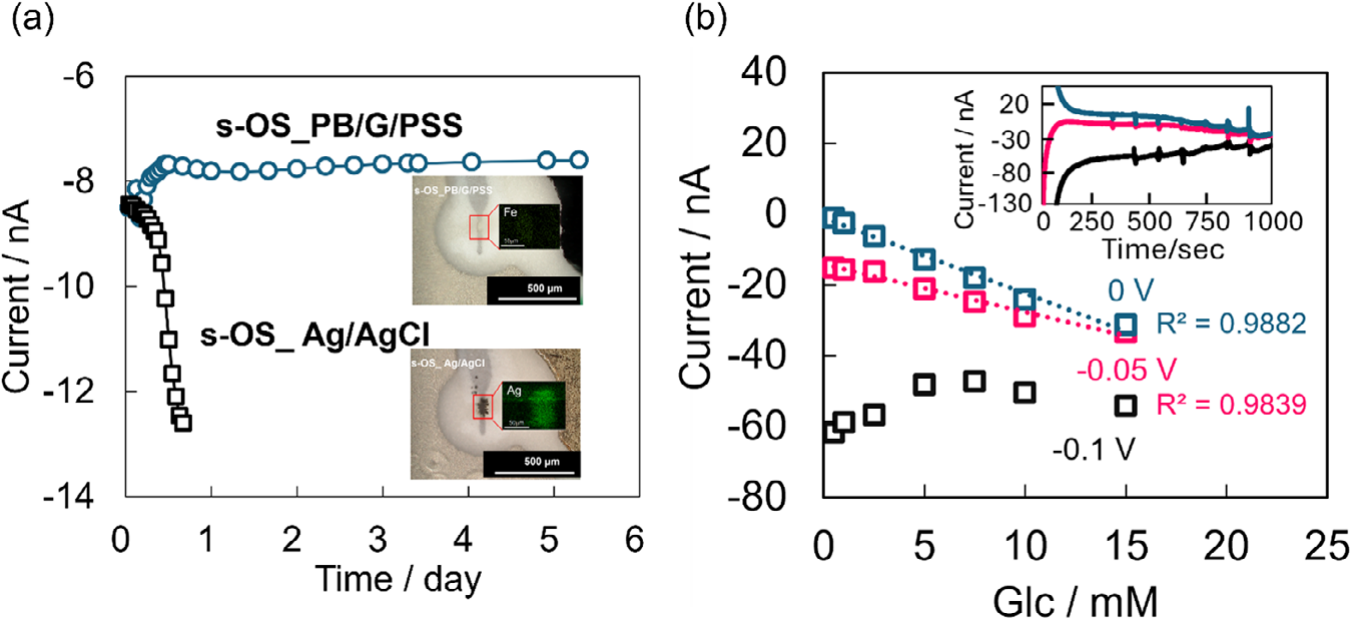
Comparison of electrochemical performance of PB/G/PSS‐based electrodes as (a) a contamination‐free reference electrode and (b) a working electrode for glucose sensing. (a) Adapted with permission [38]. Copyright 2024, American Chemical Society. (b) Adapted with permission [51]. Copyright 2025, American Chemical Society.

As discussed above, the advent of new analytical techniques is bringing carbon materials closer to being designable materials whose structure–function relationships can be explicitly considered, rather than black‐box materials used empirically. In this section, we highlight material and electrode design strategies that integrate active sites, pore structures, particle morphology, and conductive pathways. Recent studies have been reported in which these analytical methods are used to design the structure and composite state of carbon materials according to the intended electron‐transport pathway, operating potential, and reference environment, thereby addressing problems long associated with conventional carbon materials for biosensors. As one example, Yoshida and co‐workers designed an electrode material capable of functionally supporting Prussian blue on the basis of a carbon material whose pore structure and particle morphology were controlled by a templating method, and they extended this concept to solve issues in biosensor electrodes from the perspectives of both the reference and working electrodes [38, 49, 50]. Prussian blue has long been known as a pigment, but it has also attracted attention as a functional compound with low solubility and reversible redox properties. These characteristics are advantageous for stabilizing reference electrodes and detecting hydrogen peroxide. At the same time, however, Prussian blue itself is poorly conductive and tends to become unstable near neutral pH. To exploit its function in practical sensors, a support must therefore be structurally designed not merely as a carbon carrier, but so as to simultaneously provide retention, dispersion, and electron transfer. Yoshida and co‐workers addressed this need by using silica particles as templates to prepare PB/G/PSS, in which Prussian blue was loaded inside the pores of graphene‐coated porous silica spheres (G/PSS) whose spherical particle morphology and mesoporous structure had been predetermined. G/PSS is a composite particle in which the surface of porous silica particles is coated with conductive graphene, thereby preserving the silica‐derived mesoporous structure and spherical morphology while imparting electrical conductivity to the particle surface. In other words, this material independently integrates particle morphology suitable for screen printing, a pore structure capable of retaining and dispersing Prussian blue, and a conductive graphene coating layer that serves as an electron‐transport pathway. This design enabled the functions of Prussian blue to be implemented in miniaturized electrochemical devices. As a reference electrode, it realized stable oxygen sensing while avoiding metal contamination derived from Ag/AgCl (Figure 4a) [38]; as a working‐electrode material, it enabled glucose detection via H_2_O_2_ reduction even in the presence of oxygen and ascorbic acid (Figure 4b) [51].

Yukird and co‐workers demonstrated a strategy to mitigate the self‐aggregation of graphene oxide (GO) by engineering nanosheets that facilitate the uniform dispersion of iron oxide nanoparticles [52]. Despite possessing a high surface area and versatile intermolecular interactions, GO sheets are prone to restacking and aggregation, which severely compromises their effective surface area and adsorptive capacity. To address this limitation, a composite structure was developed wherein iron oxide nanoparticles are uniformly anchored onto the GO sheets. This architecture maintains maximal surface exposure by physically preventing sheet restacking. The resulting composite not only preserves surface area and adsorption performance but also integrates active adsorption sites with efficient electron‐transport pathways and magnetic separation functionality. This multifunctional platform was subsequently utilized for high‐sensitivity pesticide detection and the visualization of analyte distribution.

At the atomic scale, Behan and co‐workers demonstrated that the effect of N doping on dopamine adsorption depends strongly on the underlying carbon nanostructure [53]. Two N‐doped carbon electrodes with comparable N contents and nearly identical distributions of graphitic and pyridinic N exhibited markedly different dopamine coverages: the less‐disordered carbon showed a sixfold increase relative to undoped carbon, whereas the more‐disordered carbon showed only a 1.9‐fold increase. DFT calculations further showed that graphitic N strengthened dopamine adsorption compared with pristine graphene. These results indicate that the beneficial chemical effect of N doping can be largely offset by structural disorder, and that dopant chemistry and carbon‐framework organization must therefore be considered together when designing the reaction field.

In addition, a hydrogen peroxide sensor based on an ordered carbonaceous framework (OCF), in which molecular design can be reflected directly in the carbon framework by using an iron porphyrin molecule as the precursor, has been reported [50]. Conventional carbon materials offer high conductivity and stability, but it is not easy to define the arrangement of active sites and the pore structure precisely at the molecular level, and their design has often depended on nonuniform, empirical approaches. By contrast, OCF is a carbon material obtained by using metallo‐porphyrin crystals bearing ethynyl groups as precursors, first fixing the framework through thermal polymerization of the ethynyl groups and then carbonizing while retaining the ordered structure of the precursor crystal. This method enables the ordered structure derived from the precursor crystal and the metal–N4 active sites to be introduced into the carbon framework as an integrated whole. Based on this design concept, Fe‐OCF was created by using an iron porphyrin with four ethynyl groups as the precursor, thereby embedding heme‐like Fe–N4 active sites into an ordered carbon framework. In this Fe‐OCF, the heme‐like Fe–N4 active sites are incorporated into a regular carbon framework, thereby combining the reactivity of a molecular catalyst with the conductivity and stability of a carbon material. As a result, the stability in water, which had been insufficient for the precursor molecule itself, was improved, while access to the active sites and electron transfer were secured, yielding a stable current response to hydrogen peroxide. Furthermore, because the active sites are immobilized in an ordered manner within the carbon framework, oxidation and reduction can be switched depending on the applied potential, and enzyme‐like bidirectional catalytic behavior was demonstrated.

Together, these studies highlight dopants and active sites as powerful design variables for tailoring carbon reaction fields. Their chemistry and configuration should be optimized according to the target analyte and sensing mechanism so that electron transfer, target–surface interactions, local concentration, catalytic or coordination chemistry, and compatibility with biomolecular recognition elements can be balanced to maximize sensing performance.

These recent studies reposition carbon materials not as mere conductive supports but as materials whose sensing functions can be engineered through the design of particle morphology, pore structure, surface state, and active‐site arrangement. In other words, the structural design of carbon materials itself determines stability, electron transport, reaction fields, and mass accessibility, and thus directly leads to the resolution of longstanding challenges. In particular, the studies on N‐doped carbon and Fe‐OCF demonstrate that dopants and active sites can serve as powerful design variables when their chemistry and configuration are tailored to the target analyte and sensing mechanism. Such optimization requires consideration not only of electron transfer but also of target–surface interactions, local concentration, catalytic or coordination chemistry, and compatibility with biomolecular recognition elements. Although the examples discussed here focus on selected systems, the same structure‐guided design concept can also be applied to a broader range of carbon materials and electrode platforms, including graphene derivatives, carbon nanotubes, carbon black, carbon nanofibers, porous and heteroatom‐doped carbons, boron‐doped diamond, laser‐induced graphene, and printed carbon electrodes. This shift indicates that carbon materials are moving away from black‐box empirical materials toward rationally designable materials based on structure–function relationships.

## 5 Transparent Carbon Electrodes for Multimodal Biosensing

Recent research on carbon materials has advanced toward overcoming the longstanding limitations of conventional carbon materials for biosensors by intentionally designing electron‐transport pathways and electrochemical environments. However, in complex matrices, such as environmental or biological samples, a single electrochemical readout often fails to distinguish between target‐molecule binding and signal fluctuations caused by interfacial instability or external interference. To address this, a multimodal sensing platform is required. In this section, we highlight electrode and signal architectures that integrate optical transparency with electrochemical functionality.

A central element for realizing such multimodal measurements is the transparent electrode, which combines electrochemical functionality with optical accessibility. A transparent electrode refers to an electrode that retains the electrical conductivity required for electrochemical measurements while simultaneously providing the optical transmittance needed for optical observation. By utilizing transparent carbon electrodes, researchers can simultaneously acquire electrochemical and optical data from the same interface [54]. For example, Takemoto and co‐workers fabricated a transparent, ultrathin, and flexible organic electrochemical transistor with visible‐light transmittance exceeding 90% and showed that it could simultaneously achieve electroencephalogram (EEG) acquisition and nitrite sensing in the worn state, together with optical blood‐flow measurement directly through the device [55]. This is an important demonstration showing that transparent electrodes allow electrical or electrochemical signal acquisition and optical observation to be carried out in parallel at the same location and on the same time axis. This integrated approach allows for the cross‐validation of signals, ensuring more accurate and reliable detection in challenging environments. In this context, multimodal sensing should be understood not merely as the coexistence of multiple signal outputs, but as a self‐validating sensing strategy based on co‐localized, orthogonal signal channels.

To extend this design concept to carbon‐material‐based biosensors, conventional carbon materials, which readily absorb visible light, must be endowed with sufficient optical transparency to ensure adequate optical access to the same interface. In addition to graphene, reduced graphene oxide (rGO) films [56], carbon nanotube (CNT) networks [57], and doped carbon materials such as boron‐doped diamond [58] have also been employed as optically transparent electrodes. Among these materials, graphene can be thinned down to the atomic‐layer level and therefore exhibit high optical transparency. Indeed, monolayer graphene absorbs only ∼2.3% of white light [59] and is also known as a conductive material with high‐mobility carriers and excellent electrical transport properties [60, 61]. Graphene's unique combination of high optical transparency and electrical conductivity makes it an ideal platform for integrated multimodal sensing, enabling simultaneous optical and electrochemical measurements at a single interface [62]. However, maintaining uniform and defect‐free graphene over large areas and introducing surface functionalities without compromising its electronic structure can be challenging. In contrast, rGO and CNT networks offer greater processability and flexibility in surface functionalization, although film heterogeneity, optical scattering, and increased resistance may need to be considered. Doped carbon materials provide additional flexibility for tuning electrical and electrochemical properties, while doping can also influence optical properties and interfacial interactions. Thus, each transparent carbon material presents a distinct balance among optical transparency, electrical transport, processability, and surface chemistry, allowing the electrode material to be selected according to the requirements of the sensing application.

As an example of such application‐oriented material selection, CVD‐grown monolayer graphene has been used as an optically transparent electrode for biosensing, where Electrochemiluminescence (ECL) produced by electrochemical excitation is efficiently collected in a transmission configuration [63]. The monolayer graphene electrode exhibited optical transmittance comparable to that of ITO together with electrochemical characteristics close to those of carbon electrodes, achieving high ECL detection efficiency in the transmission configuration. Furthermore, heterogeneous ECL immunoassays using immunomagnetic beads demonstrated detection of the tumor marker carcinoembryonic antigen. ECL in this assay originates from electrochemically generated excited states, with emission intensity reflecting local electron‐transfer activity and target binding. This co‐localized and decoupled electrochemical‐optical process enables orthogonal readouts, enhancing detection reliability in complex environments. Transparent electrodes thus enable synchronized multimodal sensing measurement, providing a more comprehensive interpretation of interfacial reactions through integrated optical and electrochemical analysis.

**FIGURE 5:**
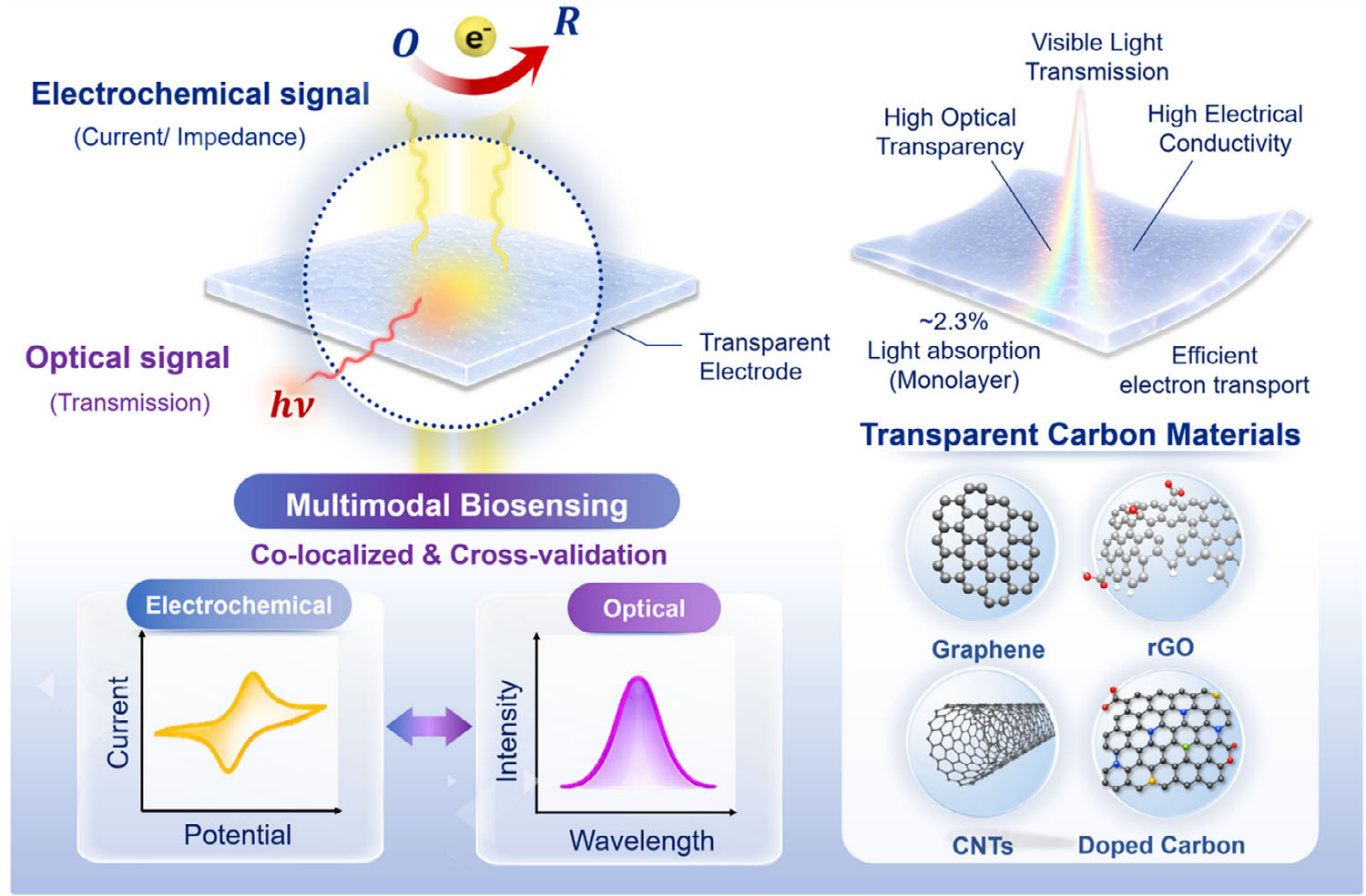
Transparent carbon electrodes enable multimodal biosensing by co‐localizing electrochemical and optical signal generation at a single electrode interface, allowing synchronized signal readout.

In this way, transparent carbon electrodes redefine multimodal biosensing as a self‐validating sensing strategy by enabling the synchronous acquisition of co‐localized, orthogonal signals from a single interface, thereby allowing a more comprehensive interpretation of interfacial reactions (Figure 5). However, multimodal systems introduce challenges such as mismatches between optical and electrochemical signals, interfacial fouling‐induced attenuation, temporal drift, and matrix‐dependent interference, necessitating signal synchronization, independent calibration of each modality, and careful interpretation of signal disagreement. In this context, transparent carbon electrodes should be understood not merely as platforms for multimodal sensing, but as reaction field components that spatially and functionally integrate electrochemical and optical processes while maintaining a reliable relationship between the two readouts. Thus, carbon materials fundamentally extend their role from conductive supports to architected interfaces that enable co‐localized, multi‐domain signal generation within a single reaction field.

## 6 From Designed Reaction Fields to Real‐World Operation

While Section 5 discusses how transparent carbon electrodes enable multimodal electrochemical–optical reaction fields, practical implementation requires maintaining these coupled reaction fields under real operating conditions. Here, we focus on device‐ and operating‐environment‐level design, including sample transport, wetting, interfacial conditions, and optical boundaries. In wearable and point‐of‐care biosensors, transport, wetting, droplet retention, optical attenuation, and fouling continuously reshape the reaction field. Therefore, these environmental and operational factors should also be regarded as design variables.

In electrochemical measurements targeting sweat, the acquired signal is known to depend not only on the concentration of the analyte molecule but also strongly on the complexity of blood‐to‐sweat partitioning, local sweat generation, evaporation, retention on the skin surface, and the state of the skin interface including sebum and the stratum corneum [64]. In addition, fluctuations in wetting and contact state at the electrode–skin interface can cause variations in interfacial impedance and response stability. Optical measurements are likewise vulnerable: droplet retention, condensation, and fogging can destabilize the readout of transmitted or reflected light and thereby undermine the reliability of simultaneous measurements. These coupled perturbations indicate that sensing performance emerges from the interplay of transport, interface, and signal‐generation processes rather than from isolated material characteristics. Thus, even when multimodal measurement itself is feasible, achieving high sensitivity and precision requires active design of the reaction field so that sample transport, molecular recognition, and interfacial or boundary conditions remain properly organized during operation.

To address these challenges, it is effective to actively design sample‐transport conditions and interfacial states. Fu and co‐workers proposed a microfluidic sweat patch in which sweat is guided to the detection region by capillary force while flow is sustained by an evaporation pump based on porous laser‐induced graphene, demonstrating that fresh sweat can be continuously supplied to the sensing region [65]. Furthermore, they established that geometric parameters‐specifically channel width, height, and length‐govern droplet velocity and transport efficiency. These findings clarify that sample introduction, retention, and renewal can be precisely regulated through structural design, transforming the sampling environment into a controlled component of the sensing system.

This perspective further emphasizes that the transport layer is not an external constraint, but a tunable design variable within the reaction field. Complementarily, interfacial states are also essential for achieving high‐sensitivity multimodal sensing. To ensure that detection limits reach clinically or environmentally significant thresholds, transparent carbon electrodes are often functionalized with optically active and highly conductive nanomaterials, such as graphene oxide (GO), gold nanoparticles (AuNPs), and quantum dots (QDs). These materials act as synergistic amplifiers, enhancing the electroactive surface area and electron‐transfer kinetics while simultaneously enabling optical functionalities such as fluorescence quenching or plasmonic enhancement. Such integration is critical for maintaining high signal‐to‐noise ratios, particularly in complex biological or environmental matrices [66, 67].

A critical component in the architecture of multimodal biosensors is the selection of the bioreceptor, or molecular recognition element. These are generally classified into enzymatic, antibody‐based, aptamer‐based, DNA‐based, or synthetic chemical reagent‐based systems. Selecting a bioreceptor that is chemically and physically compatible with both the nanomaterial modifiers and the transparent electrode properties is paramount. This synergy is the primary determinant of selectivity and analytical performance, ensuring high‐fidelity detection with minimal cross‐reactivity. By optimizing this bio‐interface, researchers can significantly reduce the risk of false‐positive or false‐negative results, a factor that is absolutely vital for the stringent requirements of clinical diagnostics and environmental regulation [68]. The electrode material, nanomaterial modifiers, and bioreceptor form an integrated interface that defines the functional core of the reaction field.

Importantly, bioreceptor integration should also be considered in relation to the structural descriptors of the carbon surface. Oxygen‐containing functional groups and edge/defect sites can provide reactive sites for covalent immobilization, whereas graphitic domains can support non‐covalent attachment through π–π interactions, for example using pyrene‐based linkers [69]. Covalent immobilization generally provides high binding stability, while non‐covalent approaches can preserve the graphitic framework and simplify functionalization [70]. However, both strategies must be optimized to maintain receptor orientation, conformational activity, and accessibility to the target. Pore accessibility is equally important because porous carbon architectures can increase receptor loading [71], but excessive confinement or high surface coverage may hinder molecular recognition and mass transport. Thus, the optimal carbon surface should not simply maximize the amount of immobilized bioreceptor, but should provide an interfacial environment that balances immobilization stability, receptor activity, target accessibility, and suppression of nonspecific adsorption.

Surface‐structure design is also an effective means of suppressing optical instability. Lecointre and co‐workers showed that on nanocone‐structured surfaces, microscale condensate droplets are less likely to become pinned, resulting in high nonwetting and antifogging properties, which suggests that micro/nanostructures can mitigate droplet retention and condensation [72]. This finding indicates that interfacial wetting and micro/nanostructure design are integral to maintaining reliable optical readout under dynamic conditions.

**FIGURE 6:**
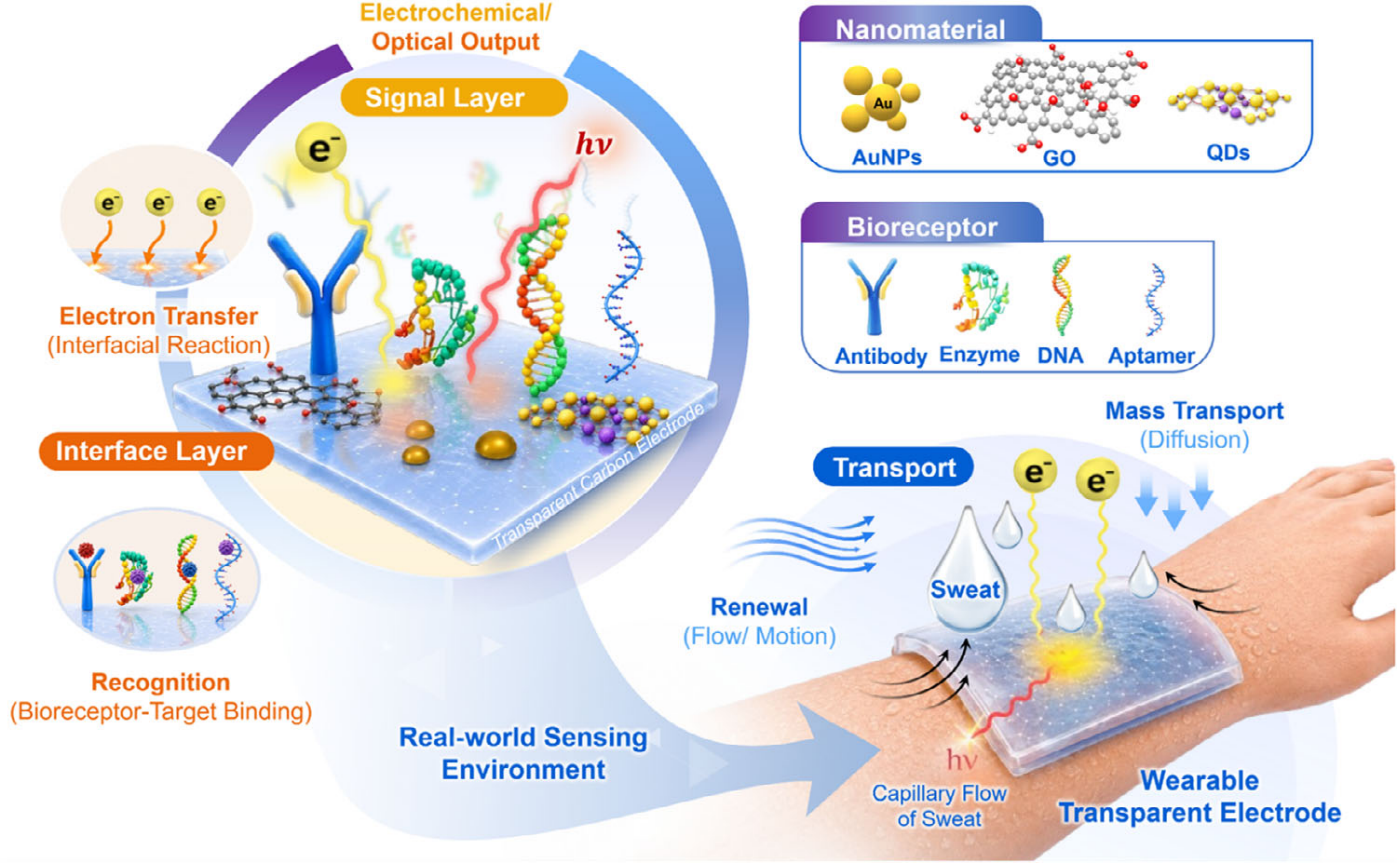
Reaction field design for real‐world biosensing, highlighting the coupled roles of transport, interfacial processes, and signal layers under dynamic conditions.

Collectively, these considerations demonstrate that reliable multimodal sensing requires the co‐design of transport, interface, and signal layers within a dynamic reaction field (Figure 6). This perspective reframes real‐world biosensing not as a materials problem, but as a reaction field engineering problem, where system performance emerges from the coordinated design of these coupled processes. In this expanded framework, carbon materials serve as the central platform for organizing transport, interfacial, and signal‐generation processes across multiple scales. Thus, carbon electrodes are no longer passive components, but hierarchically designable reaction fields governing sensing performance under real‐world operating conditions. Future advances will depend on integrating operando characterization, predictive modeling, and scalable materials design to rationally construct such dynamic reaction fields. This strategy may open new pathways not only for biosensing, but also for broader electrochemical systems requiring robust function under complex real‐world environments.

## 7 Conclusions

As discussed in this Perspective, the performance of carbon‐electrode‐based biosensors is governed by the interplay among defects, edge termination, surface functional groups, heteroatom dopants, pore architecture, electron‐transport pathways, molecular‐recognition interfaces, and electrode‐film structure. Carbon materials should therefore be regarded not simply as conductive supports, but as hierarchically designable reaction fields in which atomic structure, film architecture, interfacial chemistry, and operating conditions are considered in an integrated manner.

Future progress will depend on overcoming three major unresolved challenges: establishing quantitative causal relationships between structural and interfacial descriptors and sensing performance; developing predictive design frameworks that connect atomic‐scale structure to electrode‐ and device‐level behavior; and realizing reaction field designs that remain effective under realistic operating and scalable manufacturing conditions. Addressing these challenges will require a shift from empirical optimization toward descriptor‐based and predictive design.

A first key breakthrough will be the development of model carbon materials in which individual structural variables can be independently controlled. Defects, edge termination, surface functional groups, dopants, ordered domains, pore structure, and electrical conductivity often vary simultaneously, making it difficult to distinguish causation from correlation. Advanced characterization methods, including high‐temperature programmed desorption and solid‐state NMR, will therefore be important for resolving subtle differences in edge chemistry, surface functionality, structural ordering, and batch‐to‐batch variation. These descriptors should then be systematically correlated with electrochemical and analytical parameters, including electrochemically active surface area, charge‐transfer resistance, peak separation, capacitive background, sensitivity, limit of detection, response time, reproducibility, and stability. Such datasets will be essential for establishing transferable structure–performance relationships.

A second breakthrough will be the development of predictive multiscale models that bridge atomic structure, pore‐scale transport, electrode‐film properties, and device‐level analytical response. These models should describe how local electronic states and surface chemistry influence molecular‐recognition accessibility, mass transport, electron conduction, interfacial resistance, and signal generation across multiple length scales. Coupling atomistic, pore‐scale, film‐level, and device‐level descriptions will enable a transition from retrospective interpretation to forward design, in which the required sensing performance defines the necessary carbon structure and electrode architecture.

A third breakthrough will be the integration of operando characterization and scalable film formation into reaction field design. Under practical conditions, wetting, fouling, nonspecific adsorption, interfacial evolution, and restricted mass transport can alter both electrochemical and molecular‐recognition processes. Operando methods capable of tracking these changes together with electrochemical or optical signals will therefore be critical for validating predictive models under realistic biological and environmental conditions. At the same time, film formation and deposition history must be treated as intrinsic design variables rather than secondary processing details. Deposited mass, coating thickness and uniformity, areal coverage, roughness, wettability, binder content, adhesion, and interfacial connectivity must be controlled to preserve conductive pathways, pore accessibility, and recognition interfaces during scalable fabrication.

Together, these advances should enable carbon‐electrode biosensors to move beyond empirical material comparison toward predictive and transferable reaction field design. The decisive breakthrough will not be the identification of a single superior carbon material, but the establishment of quantitative design rules that connect atomic structure, pore architecture, electrode‐film properties, molecular‐recognition interfaces, and operating environments, while remaining valid in practical sensing and manufacturing contexts.

## Conflicts of Interest

The authors declare no conflicts of interest.

## Data Availability

The data that support the findings of this study are available from the corresponding author upon reasonable request.
